# Transcriptome analysis of two recombinant inbred lines of common bean contrasting for symbiotic nitrogen fixation

**DOI:** 10.1371/journal.pone.0172141

**Published:** 2017-02-13

**Authors:** Kelvin Kamfwa, Dongyan Zhao, James D. Kelly, Karen A. Cichy

**Affiliations:** 1 Department of Plant Sciences, University of Zambia, Lusaka, Zambia; 2 Department of Plant Soil and Microbial Sciences, Michigan State University, East Lansing, Michigan, United States of America; 3 Department of Plant Biology, Michigan State University, East Lansing, Michigan, United States of America; 4 U.S. Department of Agriculture-Agriculture Research Services, Sugarbeet and Bean Research Unit, East Lansing, Michigan, United States of America; Università Politecnica delle Marche, ITALY

## Abstract

Common bean (*Phaseolus vulgaris* L.) fixes atmospheric nitrogen (N_2_) through symbiotic nitrogen fixation (SNF) at levels lower than other grain legume crops. An understanding of the genes and molecular mechanisms underlying SNF will enable more effective strategies for the genetic improvement of SNF traits in common bean. In this study, transcriptome profiling was used to identify genes and molecular mechanisms underlying SNF differences between two common bean recombinant inbred lines that differed in their N-fixing abilities. Differential gene expression and functional enrichment analyses were performed on leaves, nodules and roots of the two lines when grown under N-fixing and non-fixing conditions. Receptor kinases, transmembrane transporters, and transcription factors were among the differentially expressed genes identified under N-fixing conditions, but not under non-fixing conditions. Genes up-regulated in the stronger nitrogen fixer, SA36, included those involved in molecular functions such as purine nucleoside binding, oxidoreductase and transmembrane receptor activities in nodules, and transport activity in roots. Transcription factors identified in this study are candidates for future work aimed at understanding the functional role of these genes in SNF. Information generated in this study will support the development of gene-based markers to accelerate genetic improvement of SNF in common bean.

## Introduction

Nitrogen (N) is the most abundant element in the atmosphere, yet it is often the most limiting element to crop productivity globally [1]. Plants belonging to family *Fabaceae* (legumes), the third largest plant family, are able to reduce atmospheric N (N_2_) to ammonia (NH_3_) through a symbiotic relationship with the soil bacteria, *Rhizobia* [2]. This relationship known as symbiotic nitrogen fixation (SNF) is a signature biological process of legumes, and takes place in nodules, which are specialized plant organs located on the roots. SNF begins with an exchange of molecular signals between the legume and rhizobia in the soil. Plant roots release molecular signal mainly in the form of flavonoids into the rhizosphere. In return, bacteria release lipo-chito-oligosaccharides (nod factor) that is perceived by plant receptor-like kinases [3]. This exchange is followed by the formation of an infection thread carrying rhizobia. The infection thread grows towards nodule primordium formed from re-programed cortical cells [4, 5]. The rhizobia infect primordium cells and move into the cytoplasm where they are surrounded by a special plant cell membrane to form an organalle-like structure called symbiosome. Inside symbiosome, rhizobia differentiate into bacteroids, which is their symbiotic form. When nodules are fully formed, the nitrogenase in bacteroids catalyzes reduction of N_2_ to NH_3_. NH_3_ is assimilated into amide glutamine. In tropical legumes such as common bean (*Phaseolus vulgaris* L.), glutamine and other amino acids are used to form purines in the cytosol of infected cells [6, 7]. Purines are oxidized into uric acid, which is transferred to uninfected cells where they are further oxidized into ureides [6]. Ureides are transported via xylem from the nodule to the roots, and then to the rest of the plant where they serve as source of N for plant nutrition. The rhizobia obtain nutrients for survival from the plant in the form of malate, a downstream photosynthetic product [8].

Over the last two decades, our understanding of genetic and molecular mechanisms involved in SNF has advanced. These advances have focused largely on genetic and genomic studies of the two model forage legume species, *Medicago truncatula* and *Lotus japonicus*. Genetic studies primarily using mutants with varying phenotypes for N-fixation such as lack of nodulation, hypernodulation, and ineffective nodules, have been used to identify genes involved in the establishment of SNF including formation and functioning of the nodules [9–13]. Some of the transcription factors (TFs) that regulate expression of genes involved in SNF have also been identified [14, 15]. In addition, key molecular mechanisms, biological processes, and pathways involved in SNF including signal transduction, carbohydrate metabolism, and purine biosynthesis have been identified [6, 16]. Transcriptome analyses in *M*. *truncatula* and *L*. *japonicus* have previously been used to gain insights into global gene expression and molecular mechanisms involved in SNF, especially the early stages of nodulation [17–22]. These transcriptomic studies have revealed a complex molecular architecture of SNF involving several genes, molecular mechanisms and pathways. Though genetic and transcriptomic studies have provided valuable information on the molecular genetics of nodulation, our understanding of genes and molecular mechanisms that play a significant role in determining genetic variability of SNF in plants with mature functioning nodules is still lacking.

Common bean is a staple crop for millions of people in East Africa and Latin America [23]. Although common bean is considered poor in SNF when compared to other economically important grain legumes such as soybean (*Glycine max*), significant genetic variability for SNF exists within common bean [24]. Effective exploitation of this variability for genetic improvement of SNF requires an understanding of genes and molecular mechanisms underlying SNF. Within common bean, studies aimed at understanding the molecular and genetic basis of SNF variability have been limited to quantitative trait loci (QTL) mapping studies and recently genome-wide association studies (GWAS) [24]. In the current study, transcriptome profiling was used to identify genes and molecular mechanisms underlying SNF differences between two common bean recombinant inbred lines, named SA36 and SA118.

## Materials and methods

### Plant materials

Two F_4:5_ recombinant inbred lines (RILs), SA36 and SA118 of common bean were used in the current study. SA36 and SA118 were chosen from a bi-parental mapping population of 213 RILs derived from a cross of Solwezi and AO-1012-29-3-3A, two Andean parents with contrasting SNF phenotypes. Solwezi is a landrace that is widely grown in Zambia. It has an indeterminate growth and large round red mottled seed type. AO-1012-29-3-3A is determinate, red kidney breeding line developed at University of Puerto Rico with resistance to seed weevils (*Acanthoscelides obtectus*) [25]. Evaluation for SNF in the greenhouse (GH) at Michigan State University (MSU) of five genotypes grown in Zambia and AO-1012-29-3-3A, showed Solwezi to be superior to AO-1012-29-3-3A in SNF. A population of 213 F_4:5_ RILs was developed from a cross of Solwezi and AO-1012-29-3-3A using single seed descent, and evaluated for SNF in the GH at MSU. Among these 213 RILs, SA36 and SA118 showed contrasting SNF phenotypes, but had similar seed type (red kidneys), growth habit (determinate) and number of days to flower (both flower at 38 days after planting). In GH evaluations, SA36 fixed more N and had higher nodule dry weight than SA118, as described in the results section.

### Growing conditions

SA36 and SA118 were grown under N fixing and non-fixing conditions in 4-liter plastic pots filled with perlite and vermiculite in a 2:1 (v/v) ratio in the GH at MSU, East Lansing, Michigan, USA in 2015. Three replications were used per growing condition. Under the non-fixing condition, 20 g of ‘Osmocot’ fertilizer (14% nitrogen, 14% phosphorus, 14% potassium) was applied to pots and thoroughly mixed with perlite and vermiculite before planting. A second 40 g of ‘Osmocot’ fertilizer (5.6 g of N) was applied to the two seedlings at trifoliate stage in each pot. High rates of N fertilizer application suppress nodulation and N fixation [26]. In addition, a nutrient solution of micronutrients was applied to ensure normal growth. SA36 and SA118 grown under non-fixing condition served as controls to the N fixing genotypes for identifying differentially expressed genes (DEGs) between SA36 and SA118 whose differential expression status were restricted to SNF for a respective tissue. Before planting, seeds were sterilized in sodium hypochlorite and then rinsed in distilled water. For plantings under fixing condition, rinsed seeds were inoculated with *Rhizobium tropici* strain *CIAT899* [27] by submerging them for two minutes in a broth culture of rhizobia made from yeast extract manitol media [28]. Inoculated and un-inoculated seeds were planted at a rate of two seeds per pot. All pots were watered with water until seeds germinated (eight days after planting), at which point, N-free nutrient solution [29] was applied to plants under N-fixing conditions while water was applied to plants growing under non-fixing conditions. To ensure effective nodulation, a second inoculation was made at germination by applying 1 ml of *CIAT899* broth to each pot of plants grown under fixing condition. Nutrient solution and water applications continued up to flowering (38 days) when samples for RNA extraction and nodule dry weight, shoot dry weight, and total N fixed were collected. Throughout the experiment, 13 hours of supplemental light per day was provided, and temperature was maintained between 23^°^C to 25^°^C in the GH. We chose to collect samples at flowering stage because at this stage the nodules are fully developed and functional. The rate of SNF peaks at flowering and declines afterwards because the pods that begin to form become a major sink for photo-assimilates, which reduces assimilates partitioned to nodules [30]. Several previous studies have focused on identifying genes involved in early stages of SNF, i.e., nodule formation [19, 21, 22] while studies focused on later stages of SNF, i.e., when nodules are fully formed, are limited. By focusing on the flowering stage, this study will provide valuable insights into genes important to explaining SNF variability at an identifiable developmental stage when SNF rates are maximal.

### Evaluation of SA36 and SA118 for SNF and related traits

To assess the SNF phenotypes of SA36 and SA118, replicated plants were harvested and separated into roots, shoot and nodules for plants grown under N-fixing condition, and into roots and shoot for plants grown under non-fixing conditions. These samples were oven-dried at 60^°^C for 72 h, and weighed to obtain shoot and nodule dry weights. The shoot tissue was ground and sent for N concentration analysis to A & L Great Lakes Laboratories, Fort Wayne, Indiana, USA. The amount of N fixed per plant for plants growing under N-fixing condition was computed as a product of N concentration in the shoot and shoot dry weight.

### Total RNA isolation, cDNA library construction, and sequencing

At flowering, leaf, nodule and root tissues were collected from N-fixing plants while only leaf and root tissues were collected from the non-fixing plants as neither SA36 nor SA118 formed nodules under these conditions. Three biological replicates of leaf, nodule and root tissues of SA36 and SA118 per condition were used. In total, 30 samples were collected, flash frozen in liquid N, and stored under -80^°^C prior to total RNA extraction. Total RNA was extracted using the TRIzol kit (Invitrogen, Carlsbad, CA, USA) following the manufacturer’s protocol and a DNAase Qiagen kit was used to remove any DNA. A spectrophotometer NanoDrop 2000 (Thermo Fisher Scientific, Waltham, MA, USA) was used to measure total RNA concentration and purity. To check the integrity of the total RNA, we used the Biological analyzer Agilent 2100 (Agilent, Santa Clara, CA, USA). Thirty mRNA-seq libraries were prepared at the Genomics Research Technology Support Facility (RTSF) at Michigan State University using the Illumina TruSeq Stranded mRNA Library preparation kit (Illumina, San Diego, CA, USA) following the manufacturer’s instructions. Libraries were pooled for multiplexed sequencing at RTSF using an Illumina HiSeq 2500 to generate single end (SE) reads of 50 nt. The raw transcriptome sequences were deposited in the NCBI SRA database (BioProject Accession number: PRJNA322335).

### Bioinformatics analyses

Read quality was assessed using FastQC [31]. Adapters were removed using Cutadapt version 1.8.1 [32] and only reads greater than 30 nt were retained. The *P*. *vulgaris* v1.0 reference genome [33] was indexed using Bowtie2 version 2.2.3 [34] and cleaned reads were aligned to the *P*. *vulgaris* v1.0 genome using TopHat2 version 2.0.14 [35] allowing a maximum of two mismatches. The minimum and maximum intron size was set to 4 bp and 11 kbp, respectively. All other parameters for TopHat were used at default settings. To determine the expression status of a gene, we used Cufflinks version 2.2.1 [36] and calculated normalized gene expression levels reported as fragments per kilobase pair of exon model per million fragments mapped (FPKM). A gene was considered expressed if its FPKM 95% confidence interval lower boundary was greater than zero.

#### Identification of DEGs and enriched molecular functions

The number of reads that mapped to a gene were counted using htseq-count from the HTSeq.py python package [37]. Gene pair-wise differential expression analysis was done using DESeq2 R package on read count values normalized to the effective library size [38]. A gene was identified as differently expressed based on false discovery rate (FDR) < 0.01 (Benjamini–Hochberg correction) [38]. DEGs were filtered further for fold expression change, and only genes with absolute Log_2_ fold-change (|Log_2_FC|) ≥ 2 were retained for downstream analyses. In this study, we focused on genes whose differential expression status was restricted to SNF fixing conditions. We hypothesized that genes with differential expression restricted to the fixing conditions would be informative as to the molecular genetic basis of the contrasting SNF phenotypes between SA36 and SA118. To identify genes in leaves or roots whose expression status was restricted to SNF, we followed two steps. First, we identified genes differentially expressed in the same tissue type between SA36 and SA118 under fixing conditions and then under non-fixing conditions. Second, we did not consider the genes that were differentially expressed under both fixing and non-fixing conditions. The final list represented genes whose differential expression status was hypothesized to be associated with SNF for a particular tissue type. For the nodules, all genes that were differentially expressed between SA36 and SA118 were presumed to be associated with SNF as no nodules formed under non-fixing conditions.

To gain insights into possible molecular mechanisms underlying the contrasting SNF phenotypes of SA36 and SA118, gene ontology (GO) term [39] enrichment analysis of DEGs (with |Log_2_FC| ≥ 2) was conducted. The singular enrichment analysis tool from AgriGO [40] was used with GO annotations from *P*. *vulgaris* v1.0 reference genome [33]. The singular enrichment analysis was done using Fisher’s test and significance threshold of FDR<0.05. To demonstrate the usefulness of the transcriptome data generated in the current study for developing gene-based markers that can be used to indirectly select for improved SNF in common bean, we called single nucleotide polymorphisms (SNPs) in the coding sequence of genes that were differentially expressed in leaf, root and nodules between SA36 and SA118 using SAMtools version 1.2 [41] and BCFtools version 1.2 [42].

## Results

### Responses of SA36 and SA118 to N fertilizer and rhizobia inoculation

**Fig 1:**
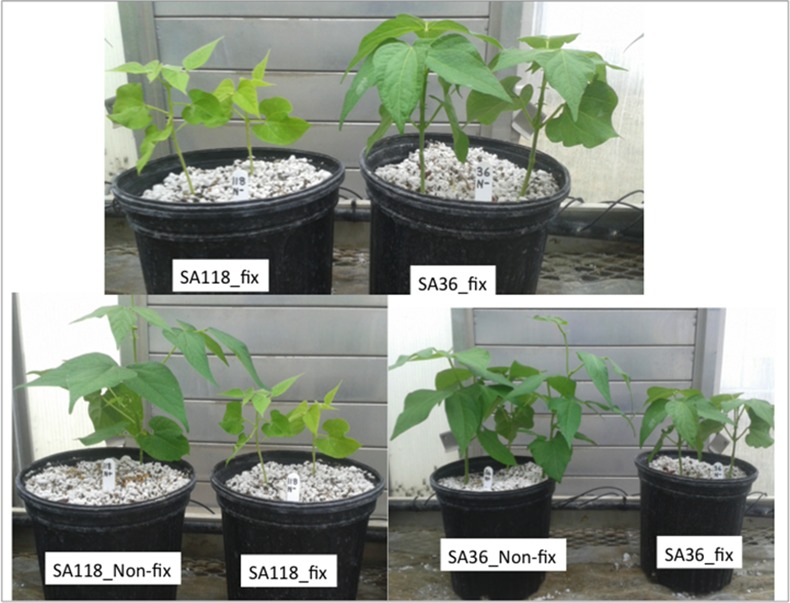
Growth characteristic of SA36 and SA118 under fixing and non-fixing condition grown in the greenhouse.

**Fig 2:**
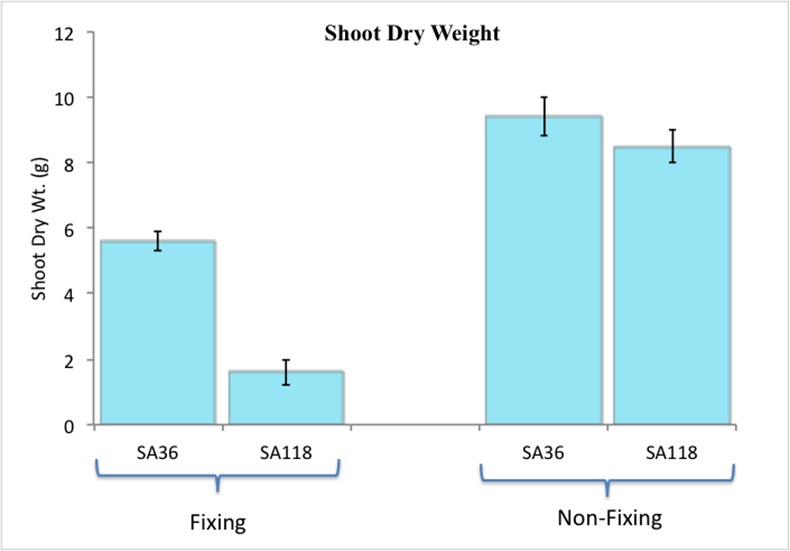
Differences in shoot dry weight (per plant) between SA36 and SA118 grown under nitrogen fixing and non-fixing conditions.

**Fig 3:**
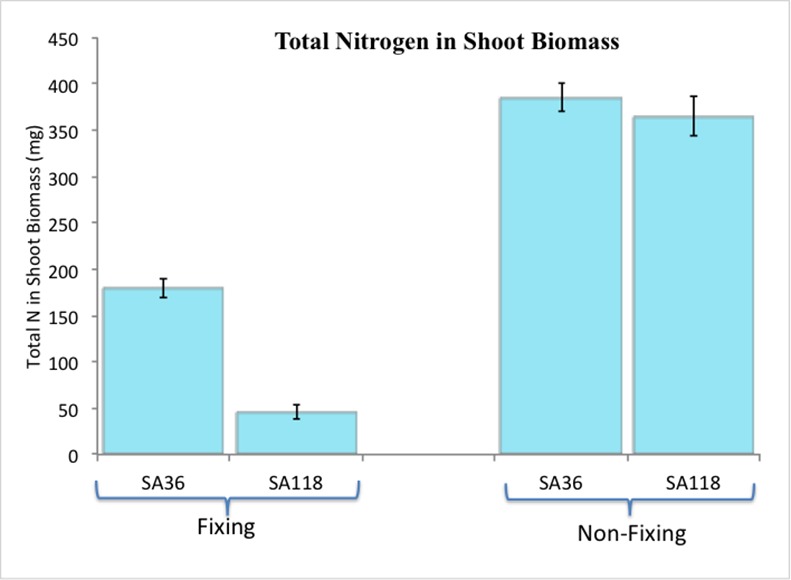
Differences in total nitrogen in shoot biomass (per plant) between SA36 and SA118 grown under nitrogen fixing and non-fixing conditions.

**Fig 4:**
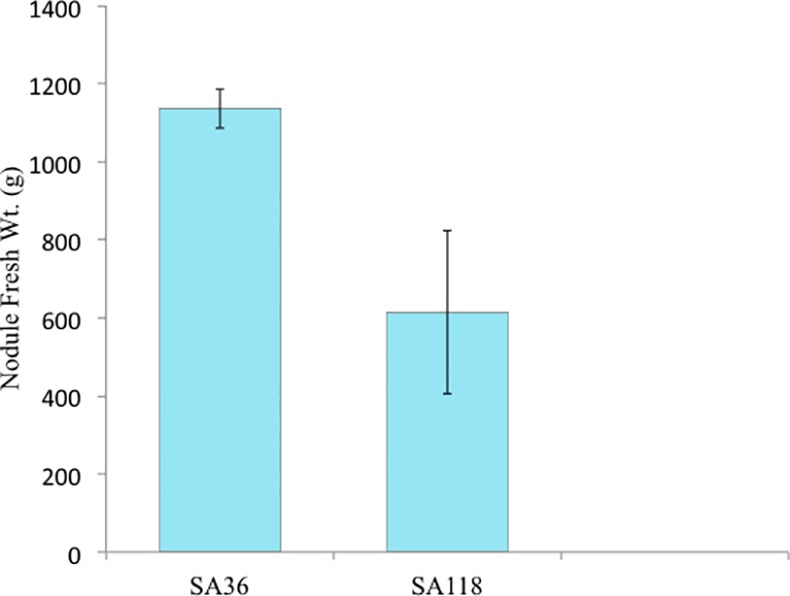
Nodule fresh weight (per plant) difference between SA36 and SA118 grown under nitrogen fixing conditions.

At flowering, both SA36 and SA118 had fully developed nodules under N-fixing conditions, but under the non-fixing conditions neither RIL formed nodules. Major differences in shoot dry weight between SA36 and SA118 were observed under the fixing conditions but not under non-fixing conditions (Fig 1). Under N-fixing conditions, the shoot dry weight for SA36 was 5.6 g plant^-1^ compared to 1.6 g plant^-1^ for SA118 (Fig 2). Under non-fixing conditions, SA36 and SA118 weighed 9.4 g plant^-1^ and 8.5 g plant^-1^, respectively (Fig 2). In terms of total N fixed per plant, which was computed as a product of shoot dry weight and N% in the shoot, SA36 was superior to SA118. SA36 fixed 179 mg plant^-1^ N, which was significantly higher than 46 mg plant^-1^ N fixed by SA118 (Fig 3). However, under non-fixing conditions, the total N in shoot dry biomass for SA36 and SA118 were similar, with 385 mg plant^-1^ N for SA36, and 365 mg plant ^-1^ N for SA118 (Fig 3). SA36 was also superior to SA118 in nodule fresh weight. The nodule fresh weight for SA36 was 1136 mg plant^-1^ compared to 615 mg plant^-1^ for SA118 (Fig 4).

### Transcriptome analyses

A total of 861 M 50 nt SE reads were generated from 30 RNA-seq libraries of leaf, root and nodule tissues of SA36 and SA118 grown under N-fixing and non-fixing conditions with three replications. The number of reads per library ranged from 19.8 M to 41.7 M with an average of 28.7 M (S1 Table). Per base quality scores for all of the libraries was greater than 25. After removing adapters, and discarding reads with less than 30 nt, reads per library ranged from 19.7 M to 41.4 M with an average of 28.4 M (S1 Table). The average percentage of mapped reads in the 30 libraries was 97.1% (S1 Table). The average percentage of uniquely mapped reads of the total mapped reads was 94.6% (S1 Table). Overall, these metrics suggest that our libraries are of high quality and provide a robust representation of transcripts in the samples within this study. Pearson product-moment correlation analyses of normalized read counts were conducted using PROC CORR in SAS 9.3 [43] to determine quality of replicates and library integrity. Average correlation coefficients under fixing condition among replicates within tissue type were 0.99, 0.99 and 0.94 for leaf, root and nodule, respectively, for SA36 and 0.99, 0.99 and 0.84 for leaf, root and nodule, respectively, for SA118 (S2 Table).

#### Differentially expressed genes between leaves of SA36 and SA118

Under N-fixing conditions, 22,715 genes were expressed in the leaves of SA36 and SA118, representing 83.5% of the estimated 27,197 genes in *P*. *vulgaris* whereas 22,811 genes were expressed under non-fixing conditions. A total of 380 genes were differentially expressed between leaves of SA36 and SA118 under non-fixing condition. There were 59 genes that were differentially expressed between leaves of SA36 and SA118 under fixing condition, but not under non-fixing condition (S3 Table). We hypothesize that the differential expression status of these 59 genes was related to SNF. Of these 59 DEGs, 15 lacked a functional annotation in Phytozome 10.3. Among the 59 DEGs 38 were up-regulated in SA36 while 21 were up-regulated in SA118 (Table 1). Among the DEGs up-regulated in SA36, genes encoding xyloglucan:xyloglucosyl transferase involved in carbohydrate metabolism were the most represented (five out of 38 DEGs). Three genes encoding leucine rich repeat receptor-like kinases (LRR-RLK) were up-regulated in SA118 compared to one in SA36. Three genes encoding AP2, Homeobox and GT-2 TFs were up-regulated in SA36 whereas two genes encoding WRKY and MYB TFs were up-regulated in SA118 (Table 2). In GO enrichment analysis, transferase activity (transferring hexosyl groups) (GO:0016758) was the only significantly enriched molecular function of DEGs up-regulated in SA36 (Table 3), whereas, there were no enriched molecular functions of DEGs up-regulated in leaves of SA118.

**Table 1:** Number of differentially expressed genes in leaves, roots and nodules between SA36 and SA118. These numbers represent genes that were differentially expressed between SA36 and SA118 under N-fixing conditions, but not under non-fixing conditions.

Tissue Organ		Up-regulated (|Log_2_ (FC)| ≥ 2)	
Comparison	DEGs (|Log_2_ (FC)| ≥ 2)	SA36	SA118
SA36 Leaf vs. SA118 Leaf	59	38	21
SA36 Root vs. SA118 Root	121	86	35
SA36 Nodule vs. SA118 Nodule	558	147	411

DEGs, differentially expressed genes: |Log_2_ (FC)| ≥ 2, absolute logarithmic fold change in expression greater or equal.

**Table 2:** Differentially expressed transcription factors. These are transcription factors with differential expression between SA36 and SA118 in leaf, root and nodule under N-fixing condition, but were not differentially expressed under non-fixing conditions.

Gene Identifier	Chr. (Position in bp)	Transcription Factor	Log_2_FC	Adj. P
**Leaf: Up-regulated in SA36**				
Phvul.004G122000	Pv04 (39326716–39327951)	AP2	2.5	0.0070
Phvul.001G187000	Pv01 (45258083–45261720)	GT-2	2.2	0.0056
Phvul.010G148700	Pv10 (41934612–41940511)	Homeobox	2.0	1.2E-08
**Leaf: Up-regulated in SA118**				
Phvul.005G018500	Pv05 (1604423–1605864)	MYB	-2.2	1.1E-06
Phvul.006G074600	Pv06 (19393601–19396850)	WRKY	-3.2	0.0068
**Root: Up-regulated in SA36**				
Phvul.002G292600	Pv02 (45587489–45590225)	MYB	2.6	3.2E-08
Phvul.007G208400	Pv07 (44697797–44699909)	MYB	2.1	0.0010
Phvul.004G171200	Pv04 (45277672–45279263)	MYB	2.0	0.0045
**Root: Up-regulated in SA118**				
Phvul.006G188900	Pv06 (29705815–29707591)	NAM	-2.3	0.0044
Phvul.001G044500	Pv01 (4680371–4681060)	AP2	-4.2	5.0E-05
Phvul.006G106100	Pv06 (22259920–22260531)	AP2	-2.6	0.0032
**Nodule: Up-regulated in SA36**				
Phvul.003G094700	Pv03 (19512352–19514272)	bHLH	5.0	1.2E-12
Phvul.010G148700	Pv10 (41934612–41940511)	Homeobox	2.3	3.9E-04
Phvul.011G005800	Pv11 (430648–437018)	MADS BOX	3.3	1.9E-17
Phvul.007G048000	Pv07 (3876555–3877440)	MADS BOX	2.8	0.0093
Phvul.004G162100	Pv04 (44426684–44427426)	MBF1	3.5	5.9E-05
**Nodule: Up-regulated in SA118**				
Phvul.004G169800	Pv04 (45126736–45127899)	AP2	-5.0	1.5E-15
Phvul.010G050500	Pv10 (8020695–8021348)	AP2	-5.7	3.1E-16
Phvul.001G044500	Pv01 (4680371–4681060)	AP2	-4.1	4.4E-05
Phvul.009G196900	Pv09 (29159605–29160767)	AP2	-2.4	0.0066
Phvul.002G036000	Pv02 (3561530–3562521)	AP2	-2.8	0.0088
Phvul.010G050800	Pv10 (8082893–8083593)	AP2	-3.0	1.4E-08
Phvul.003G102500	Pv03 (25181566–25183062)	AP2	-3.2	0.0001
Phvul.003G212800	Pv03 (42804542–42805711)	AP2	-3.9	9.5E-06
Phvul.003G292400	Pv03 (51831261–51832171)	AP2	-4.0	4.0E-07
Phvul.007G273000	Pv07 (51127595–51128470)	AP2	-2.9	0.0008
Phvul.002G007500	Pv02 (860605–862788)	bHLH	-2.1	0.0056
Phvul.003G231200	Pv03 (45237056–45239851)	bHLH	-2.5	0.0005
Phvul.003G231100	Pv03 (45216543–45218543)	bHLH	-3.4	0.0003
Phvul.011G024700	Pv11 (2054940–2056988)	NAM	-2.8	2.8E-14
Phvul.009G152900	Pv09 (22214660–22216369)	NAM	-2.7	4.4E-05
Phvul.003G248500	Pv03 (47458824–47459525)	Dof	-2.4	5.9E-06
Phvul.003G212200	Pv03 (42719744–42722190)	GRAS	-2.5	0.0015
Phvul.011G109600	Pv11 (13902942–13904399)	MYB	-3.7	0.0003
Phvul.007G108500	Pv07 (13461806–13464239)	MYB	-2.1	3.0E-11
Phvul.003G232300	Pv03 (45418410–45419954)	MYB	-2.2	0.0083
Phvul.001G215100	Pv01 (47821425–47822714)	MYB	-3.3	0.0001
Phvul.007G242300	Pv07 (48190783–48192713)	MYB	-4.6	8.3E-09
Phvul.004G053600	Pv04 (6865813–6867929)	MYB	-2.5	0.0001
Phvul.009G062700	Pv09 (10947123–10947797)	MYB	-2.7	0.0001
Phvul.007G211800	Pv07 (45045204–45046968)	MYB	-2.9	0.0026
Phvul.003G173300	Pv03 (38424473–38426629)	PLATZ	-2.4	0.0021
Phvul.002G265400	Pv02 (43085670–43087004)	WRKY	-2.0	1.1E-07
Phvul.006G111700	Pv06 (22762481–22764805)	WRKY	-2.1	8.8E-07
Phvul.005G181800	Pv05 (40322573–40324669)	WRKY	-2.1	0.0009
Phvul.002G297100	Pv02 (46023368–46025419)	WRKY	-3.5	1.6E-13
Phvul.009G137500	Pv09 (20185631–20187441)	WRKY	-4.0	9.8E-10
Phvul.010G111900	Pv10 (37576223–37578860)	WRKY	-4.4	3.3E-25

Chr., chromosome; Position, is the physical position in base pair (bp); Log_2_FC, Log2 fold change in expression of SA36 over SA118; Adj. P, is the corrected P-value for FDR = 0.01

**Table 3:** Enriched molecular functions of differentially expressed genes in leaves, roots and nodules between SA36 and SA118.

GO Identifier	Molecular Function	# (Input List)	# (Ref)	P-value	FDR
**Leaf: Molecular functions of DEGs Up-regulated in SA36**					
GO:0016758	Transferase activity, transferring hexosyl groups	5	387	0.0001	0.0017
**Root: Molecular functions of DEGs Up-regulated in SA36**					
GO:0005215	Transporter activity	9	820	0.0005	0.0250
GO:0005506	Iron ion binding activity	6	642	0.0022	0.0420
**Root: Molecular functions of DEGs Up-regulated in SA118**					
GO:0016491	Oxidoreductase activity	8	1621	0.0012	0.0069
**Nodule: Molecular functions of DEGs Up-regulated in SA36**					
GO:0004888	Transmembrane receptor activity	7	129	9.4E-06	0.0005
GO:0001883	Purine nucleoside binding	25	2587	0.0027	0.0400
GO:0016491	Oxidoreductase activity	18	1626	0.0030	0.0400
**Nodule: Molecular functions of DEGs Up-regulated in SA118**					
GO:0004312	Fatty-acid synthase activity	5	15	1.5E-05	0.0025
GO:0016798	Hydrolase activity, acting on glycosyl bonds	18	420	0.0003	0.0200

GO is Gene Ontology; # (Input List) is number of genes in the input list of differentially expressed genes with this molecular function; # (Ref) is number of genes in the reference genome with this molecular function; GO categories were identified using the AgriGO Singular Enrichment Analysis; FDR, false discovery rate.

#### Differentially expressed genes between roots of SA36 and SA118

A total of 23,313 genes were expressed in roots of SA36 and SA118 under fixing conditions, representing 86% of the estimated genes in *P*. *vulgaris*. Under non-fixing condition, 23,289 genes were expressed in roots of SA36 and SA118. A total of 2529 genes were differentially expressed between roots of SA36 and SA118 under non-fixing condition. There were 121 genes that were differentially expressed between roots of SA36 and SA118 under fixing condition, but not under non-fixing condition (S4 Table). We hypothesize that the differential expression status of these 121 genes was related to SNF activities in the roots, and possibly contributing to SNF phenotypic differences between SA36 and SA118. Out of the 121 DEGs, 35 did not have functional annotation on Phytozome 10.3. Of the 121 DEGs, 86 were up-regulated in SA36 compared to 35 up-regulated in SA118 (Table 1; S4 Table). Among the 86 DEGs up-regulated in SA36, eight encode transporter proteins, which was the most represented group, including a MFS transporter (*Phvul*.*008G011700*), an aquaporin (*Phvul*.*011G067200*), ABC transporters (*Phvul*.*002G176600*, *Phvul*.*007G078000*, *Phvul*.*003G283900*), zinc/iron transporters (*Phvul*.*006G001000* and *Phvul*.*006G003300*) and a sugar transporter (*Phvul*.*009G030800*). In contrast, there were no genes encoding transporter proteins among the 36 genes up-regulated in SA118. Four genes (*Phvul*.*011G068300*, *Phvul*.*007G238100*, *Phvul*.*007G238200*, and *Phvul*.*008G018700*) encoding nucleoporins were up-regulated in SA36 in contrast to SA118 where no nucleoporins were up-regulated. Three genes, all encoding MYB TFs were up-regulated in SA36 while two genes encoding NAM and AP2 TFs were up-regulated in SA118 (Table 2). The GO term enrichment analysis of DEGs between roots of SA36 and SA118 identified transporter activity (GO:0005215) and iron ion binding (GO:0005506) as enriched molecular functions of DEGs up-regulated in SA36 (Table 3). For DEGs up-regulated in SA118, oxidoreductase (GO:0016491) activity was the only enriched molecular function observed (Table 3).

#### Differentially expressed genes between nodules of SA118 and SA36

A total of 22,066 genes were expressed in nodules of SA36 and SA118, representing 81.1% of the estimated genes in *P*. *vulgaris*. There were 558 DEGs between nodules of SA36 and SA118 (S5 Table). Of these 558 DEGs, 131 did not have functional annotation on Phytozome 10.3. Out of 558 DEGs, 147 were up-regulated in SA36 while 411 were up-regulated in SA118 (S5 Table). Genes that encode transporter proteins, LRR-RLKs and TFs were among the 147 DEGs up-regulated in SA36 (S5 Table).

A total of ten genes encoding transporter proteins were up-regulated in SA36 nodules including *Phvul*.*011G196900* (EamA-like transporter), *Phvul*.*001G028700* (xanthine-uracil permease), *Phvul*.*007G025900* (malate transporter), *Phvul*.*007G244600* (Nodulin-like monocarboxylate transporter) (S5 Table). *Phvul*.*002G214100* encoding glutamine synthatase involved in fixed N assimilation was among DEGs up-regulated in SA36. In contrast, only three transporter genes were up-regulated in SA118 nodules.

**Fig 5:**
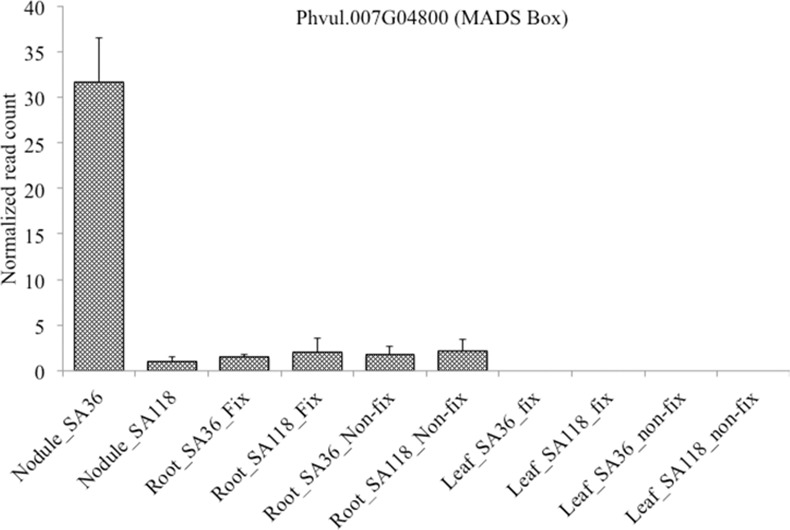
Relative expression of *Phvul*.*007G048000* (MADS BOX transcription factor) in leaves, roots and nodules of SA36 and SA118 grown under nitrogen fixing and non-fixing condition. Relative gene expression is presented using read count. Read count is number of reads (average of three replications) aligned to the gene after normalizing for total number of reads mapped for each library using HTSeq.

**Fig 6:**
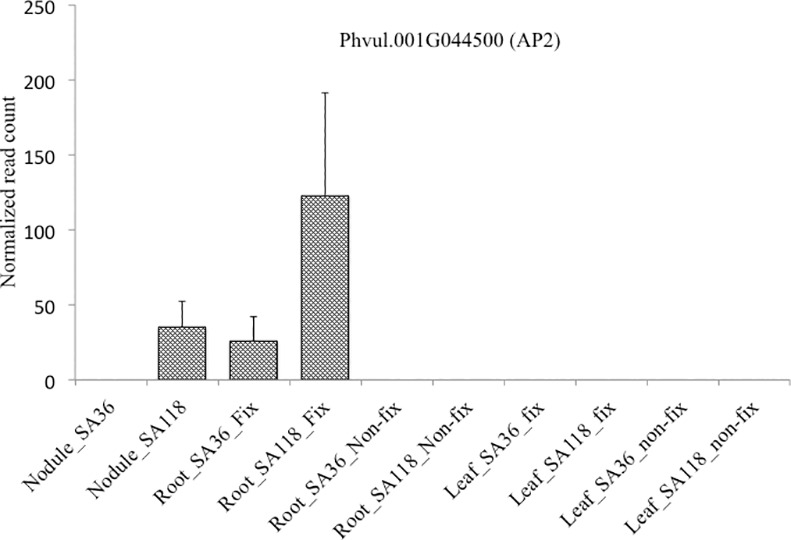
Relative expression of *Phvul*.*001G044500* (AP2 transcription factor) in leaves, roots and nodules of SA36 and SA118 grown under nitrogen fixing and non-fixing conditions. Relative gene expression is presented using read count. Read count is number of reads (average of three replications) aligned to the gene after normalizing for total number of reads mapped for each library using HTSeq.

A total of 36 genes encoding TFs were differentially expressed in nodules between SA36 and SA118 (Table 2). Of the 36 TFs genes, five genes encoding bHLH, MBF1, MADS-box and homeobox TFs were up-regulated in SA36. Among these five, *Phvul*.*007G048000* encoding MADS BOX was only expressed in nodules and roots (Fig 5). In the roots *Phvul*.*007G04800* was weakly expressed in both SA36 and SA118 (Fig 5). In SA118, 31 genes encoding AP2 (10), MYB (8), WRKY (6), bHLH (3), NAM (2), PLATZ (1), Dof (1) and GRAS (1) TFs were up-regulated. Among the AP2 encoding genes up-regulated in SA118, *Phvul*.*001G044500* was only expressed in nodules and roots under fixing condition (Fig 6).

The GO term enrichment analysis of DEGs between nodules of SA36 and SA118 identified purine ribonucleotide binding (GO:0001883), transmembrane receptor activity (GO:0004888) and oxidoreductase activity (GO:0016491) as significantly enriched molecular functions of genes up-regulated in SA36 (Table 3). Significantly enriched molecular functions of DEGs up-regulated in SA118 included fatty-acid synthase activity (GO:0004312) and hydrolase activity (GO:0016798).

#### Single nucleotide polymorphisms in differentially expressed genes

A total of 1464 SNPs were called in the DEGs. SNPs were present in 32 of the 59 DEGs in leaf tissue (113 SNPs in total) (S6 Table), 60 of the 121 DEGs in roots contained SNPs (287 SNPs in total) (S7 Table) and 271 of the 558 DEGs in nodules contained SNPs (1120 SNPs in total) (S8 Table).

## Discussion

Effective utilization of existing genetic variability in common bean for improving SNF requires an understanding of underlying genes and molecular mechanisms. This study explored the utility of transcriptome profiling to further our understanding of molecular genetic differences underlying contrasting SNF phenotypes of two RILs SA36 and SA118. Though transcriptome profiling for SNF has been conducted in two model forage legume plants, *M*. *truncatula* and *L*. *japonicus* using wild type and mutants that differ in N-fixation, the application of basic knowledge from these studies to improve SNF of grain legumes has been limited. By using *P*. *vulgaris* RILs with breeding value, our study has potential to bridge the gap between basic studies and applied use of knowledge generated from basic studies to enhance SNF of common bean, a staple food crop for millions of people in Africa and Latin America.

We compared the phenotypic performance of SA36 and SA118 under N-fixing and non-fixing conditions in the GH. Both SA36 and SA118 flower at 38 days after planting. At flowering, SA36 was superior to SA118 in shoot dry weight, nodule dry weight, and total amount of N fixed under N fixing conditions. However, shoot dry weight, and total N in shoot biomass under non-fixing conditions were similar between SA36 and SA118. These results suggest that observed differences in shoot and root dry weights between SA36 and SA118 under fixing conditions resulted from differences in SNF rates, and that under non-fixing conditions with an optimal source of soil N, SA36 and SA118 have similar capacities to accumulate shoot biomass and N. These phenotypic results of similar biomass and N accumulation under non-fixing conditions yet drastically different values under N-fixing conditions provide strong support for the use of these two RILs to identify genes that control SNF genetic variability in common bean.

### DEGs between leaves for SA36 and SA118 and enriched molecular functions

This study identified several DEGs in leaves between SA36 and SA118 whose differential expression status was associated with SNF. Genes encoding proteins involved in carbohydrate metabolism were among DEGs, and the majority of these were up-regulated in SA36 (RIL with higher SNF rate). In addition, the enriched molecular function of DEGs up-regulated in SA36 was transferase activity (transferring of hexosyl groups), which is associated with carbohydrate metabolism. As leaves are the primary source of carbon for nodule metabolism, a genotype with high SNF ability is expected to have high carbohydrate metabolism activities, consistent with the higher expression of carbohydrate metabolism genes in the leaves of SA36 than SA118.

Among DEGs, one and three genes encoding LRR-RLK were up-regulated in SA36 and SA118, respectively. Receptor kinases have been implicated in local and long distance regulation of nodule development [16]. It is plausible that receptor kinases identified in the current study as differentially expressed in leaves could be involved in long distance regulation of nodule number, nodule development, or nodule functioning. Apart from the role of leaves as a source of carbon and sink for fixed N, and in long distance signaling to regulate nodulation [10], other contributions of leaves to SNF are still not well understood. Genes identified in this study as differentially expressed, and important to SNF represent candidates for future studies aimed at expanding our understanding of the additional contribution of leaves to SNF.

### DEGs between roots for SA36 and SA118 and enriched molecular functions

Carbon and N fluxes between nodules and the rest of the plant rely on transporter proteins in the roots. Consistent with this, several genes encoding transporter proteins were among 347 DEGs in roots between SA36 and SA118. The majority of these transporter genes were up-regulated in SA36 (RIL with higher SNF rate). Additionally, transporter activity was one of the enriched molecular functions of DEGs up-regulated in SA36. The transporter genes up-regulated in SA36 encode two ABC transporters, two sugar transporters, two iron transporters and an aquaporin transporter. Phvul.009G030800 encoding a transmembrane sugar transporter that was not only up-regulated in roots of SA36, was also strongly up-regulated (LogFC = 4.6) in the nodules of SA36. These results may suggest higher fluxes of carbon and other elements from the shoot to nodules, and may be N compounds from nodules to the rest of the plant in SA36 than SA118. This further suggests more available carbon and other elements for nodule metabolism and corresponding increases in SNF in SA36 than in SA118. Four genes encoding nucleoporins were up-regulated in SA36. In contrast, no genes encoding nucleoporins were up-regulated in SA118. Nucleoporins are constituents of the nuclear pore complex that mediates macromolecular transport such as mRNA and protein across the nuclear envelope [44]. Nucleoporins have been implicated in calcium spiking in roots associated with early events of nodulation. The mutant (*nup85*) with defective expression of a nucleoporin in the roots of *L*. *japonicus* was also defective in root nodule symbiosis and nod-factor induced calcium spiking [44]. Iron binding activity (GO:0005506) was the second molecular function enriched in DEGs up-regulated in SA36. Genes encoding hemopexin and hemerythrin, which binds iron were up-regulated in SA36. In addition, genes encoding iron dehydrogenase that is involved in iron metabolism were up-regulated in SA36, which had higher SNF rate than SA118. Iron is required for synthesis of iron-containing compounds essential to SNF in both the plant and rhizobia. In rhizobia, iron is required for synthesis of nitrogenase complex and is part of the FeMo co-factor required for reducing N_2_ to NH_3_. In the plant, iron is a component of the heme moiety of leghemoglobin that facilitates oxygen diffusion to respiring rhizobia under low oxygen environment needed for functioning of the rhizobia [45].

### DEGs between nodules of SA36 and SA118 and enriched molecular functions

Metabolic cooperation between rhizobia and the legume host is the basis of SNF. The plant supplies malate to rhizobia in exchange for reduced nitrogen from the rhizobia. These exchanges happen in the nodule. Therefore, metabolism and transport of carbon and N are key physiological processes of the nodule. The purine biosynthesis pathway plays a dominant role in N metabolism of tropical legumes such as common bean and soybean [6]. In these legumes, fixed N (NH^+^_4_) is first assimilated into glutamine. Through the purine pathway, the assimilated N is converted into inosine monophosphate (IMP), and after a series of oxidation and enzymatic steps, IMP is converted into ureides that are transported from the nodule into xylem vessels of roots for distribution to the rest of the plant [6, 7]. Glutamine synthatase (GS) is required for assimilation of fixed NH_4_ into glutamine (Lam et al., 1996). *Phvul*.*002G214100* that encodes glutamine synthatase (GS) was strongly up-regulated (Log_2_FC = 3.4) in SA36. It is plausible that *Phvul*.*002G214100* is one of the GS genes involved in assimilation of NH_3_. The purine nucleoside binding activity (GO:0001883) was among the enriched molecular functions of DEGs that were up-regulated in SA36. The higher oxidoreductase enzyme activity in SA36 than SA118 could have been necessary in meeting the increased oxidation reactions of converting IMP to ureides in SA36, consistent with the observed higher SNF rates for SA36 than SA118.

The transport system is a key component of the *P*. *vulgaris*-rhizobia symbiosis that handles carbon and nitrogen fluxes in the nodule. The symbiosome membrane is a critical interface of fluxes between the plant and rhizobia [46]. In addition to transport across symbiosome membrane, transport across plasma membranes plays an important role in carbon and N metabolism in the nodule [47]. In this study, several genes encoding transporter proteins were differentially expressed between SA36 and SA118. The majority of genes involved in the transportation of carbon and N compounds were up-regulated in SA36. In addition, transmembrane transport activity was among the significantly enriched molecular functions of DEGs up-regulated in SA36. *Phvul*.*011G196900* encoding an EamA-like transporter was strongly up-regulated (Log_2_FC = 3.2) in SA36. *Phvul*.*011G196900* is a homologue of *Medtr8g041390* (*MtN21/EamA-like* gene) in *M*. *truncatula* and *Glyma*.*13G189700* in soybean. In *M*. *truncatula*, *MtN21/EamA-like* was initially described as a nodulin induced during *M*. *truncatula-R*. *meliloti* symbiosis [48]. *MtN21/EamA-like* contains a metabolite transporter domain characteristic of proteins that transport amino acids such as glutamine and asparagine [49]. Once N_2_ has been fixed into NH_3_ in the symbiosome, it is exported to cell cytosol were it is first assimilated into amides glutamine and asparagine, important compounds in the SNF process [50]. The strong up-regulation of *EamA-like* transporter may suggest higher flux of glutamine in SA36 than SA118, and is consistent with the observed up-regulation of *Phvul*.*002G214100* (Glutamine synthatase) in SA36 nodules.

In tropical legumes such as common bean, ureides are important storage and transport form of fixed N. The upstream compounds for synthesis of ureides include xanthine and uric acid [6]. Xanthine is transported in the cell by xanthine-uracil permeases. *Phvul*.*001G028700*, which encodes xanthine-uracil permeases, was up-regulated in SA36, suggesting higher synthesis of ureides in SA36 than SA118. Malate supplied by the plant is the source of reduced carbon for bacteroid metabolism [51]. A malate transporter gene *Phvul*.*007G025900* was strongly up-regulated (Log_2_FC = 3.9) in SA36 compared to SA118, suggesting there could have been higher influx of malate to the bacteroids in SA36 than SA118. Overall, more transporter genes were up-regulated in nodules of SA36 than SA118, suggesting there could have been higher fluxes of carbon and N, in addition to other compounds, in the nodules of SA36 than SA118.

Receptor kinases are a key component of signal transduction, and have been implicated in local and long distance regulation of nodule development [16, 52]. Whereas the role of receptor kinases in early stages of symbiosis has been proposed, the role of receptor kinases in the functioning of mature nodules is not well understood. In the current study, transmembrane receptor kinase activity (GO:0004888) was among molecular functions significantly enriched in DEGs upregulated in nodules of SA36. A total of 21 genes encoding LRR-RLK’s were up-regulated in SA118 compared to three up-regulated in SA36. The differentially expressed LRR-RLK genes identified in the current study are strong candidates for future studies aimed at characterizing the functional role of LRR-RLK genes in mature nodule functioning.

The functional role of most TFs in legumes, particularly in SNF, a signature biological process of legumes remains unknown [15]. In a developmentally complex process such as SNF, which involves expression of several genes in many pathways, TFs are expected to play a leading role in coordinating expression of these genes. Some of the TFs involved in the early stage of symbiosis including ethylene response, GRAS, bZIP, C_2_H_2_ and AP2-ERFBP TFs have been identified in previous studies [14, 53, 54, 55]. However, knowledge of TFs involved in the functioning of mature nodules, which may explain contrasting SNF phenotypes of common bean, is limited. In this study, genes encoding TFs that may be important to functioning of mature nodules, and possibly contributing to molecular genetic differences underlying the contrasting SNF phenotypes of SA118 and SA36 were identified. Among the 558 DEGs in the nodules, 36 encode TFs. Genes in *M*. *truncatula*, *L*. *japonicus* and *G*. *max* belonging to some of the TF families identified as having differentially expressed in the current study have previously been implicated in nodule development and functioning. Among the 36 TF genes differentially expressed between nodules of SA36 and SA118, *Phvul*.*007G048000* and *Phvul*.*001G044500* were particularly interesting because of their tissue specific expression patterns. *Phvul*.*007G048000* encodes a MADS box TF, and showed a 2.8 fold increase in expression in SA36 (RIL with higher SNF rate) over SA118. Interestingly, *Phvul*.*007G048000* showed no evidence of expression in leaves, and was weakly expressed in roots under both fixing and non-fixing conditions (Fig 5). This restricted expression pattern of *Phvul*.*007G048000* is consistent with a previous study, which reported that among seven diverse tissue types of common bean, *Phvul*.*007G048000* was only expressed in nodule tissue [56]. The current study provides further support to restricted tissue expression of *Phvul*.*007G048000*, but more importantly it has shown that increased expression levels of *Phvul*.*007G048000* a MADS box TF may be associated with higher SNF rate in SA36 than SA118. The genomic location of *Phvul*.*007G048000* (3,876,555 bp– 3,877,440 bp) is within the same region (3,466,123 bp– 4,742,067 bp) where a significant SNPs for SNF in a common bean Andean diversity panel was identified in GWAS evaluated under GH and field conditions [24]. Based on results of the current study and the previous GWAS, *Phvul*.*007G048000* a MADS box TF is an excellent candidate for genetic improvement in the *P*. *vulgaris-rhizobia* symbiosis. Being a TF with nodule specific expression makes *Phvul*.*007G048000* a better target for genetic improvement as it may be responsible for coordinated expression of several genes only in the nodule. Interestingly, there were five SNPs within *Phvul*.*007G048000* (S8 Table), suggesting that molecular markers can be developed for this gene for use in marker-assisted breeding for enhanced SNF rate in common bean. Among TFs up-regulated in SA118 nodules, *Phvul*.*001G044500* which encodes an AP2 TF was strongly up-regulated in SA118 (RIL with lower SNF rate) than in SA36 (Log_2_FC = 4.1). In addition, *Phvul*.*001G044500* showed significantly higher expression levels in the roots of SA118 than SA36 (Log_2_FC = 4.2) under N-fixing conditions. However, *Phvul*.*001G044500* showed no evidence of expression in roots under non-fixing conditions or in leaves under either fixing or non-fixing conditions (Fig 6). Results of this study suggest that increased expression of *Phvul*.*001G044500* AP2 TF may be associated with reduced SNF rates. In addition to *Phvul*.*001G044500*, nine other AP2 encoding genes were up-regulated in SA118. In contrast, there was no AP2 encoding gene up-regulated in SA36 (Table 2). This result provides further support for possible relationship between increased AP2 TFs expression and lower SNF rate in SA118 than SA36. Nova-Franco et al. [57] postulated that an AP2 TF (Phvul.005G138300) transcriptionally activate genes related to nodule senescence in common bean. Though this particular AP2 TF gene was not differentially expressed between nodules of SA36 and SA118 in the current study, it provides some useful insights into the possible role of ten AP2 TFs up-regulated in SA118. Five genes encoding bHLH TFs were differentially expressed in nodules between SA36 and SA118, with two and three up-regulated in SA36 and SA118, respectively. Of the two up-regulated in SA36, *Phvul*.*002G216700* is homologous to *Glyma*.*15G061400* (*GmbHLHm1*) in soybean, and *Medtr2g010450* (*MtbHLH1*) in *M*. *truncatula* (http://www.phytozome.org). Interestingly, *Phvul*.*002G216700* and *Medtr2g010450* (*MtbHLH1*) seem to have some similarities in tissue expression patterns. In the current study, *Phvul*.*002G216700* was not expressed in leaves under either N-fixing and non-fixing conditions, but was expressed in nodules and roots, which is similar to reported restricted expression of its homolog *Medtr2g010450* (*MtbHLH1*) to roots and nodules [58]. Recent functional studies demonstrated the importance of *GmbHLHm1* and *MtbHLH1* in nodule development and functioning. Soybean plants that lost *GmbHLHm1* activity showed a significant reduction in nodule number, nodule fitness and development [59]. In *M*. *truncatula*, a transgenic plant with impaired *MtbHLH1* expression produced nodules with vascular defects and exhibited poor nutrient exchanges between nodules and roots [58]. In addition, *MtbHLH1* was postulated to regulate asparagine synthase gene [58], an enzyme required for assimilation of fixed N. TF families were identified in the current study whose role in nodule development and functioning has been documented previously. Intriguingly, MBF-1, PLATZ and GT-2 TFs with no previously reported role in mature nodule functioning have also been identified as differentially expressed between mature nodules of SA36 and SA118 RILs.

One of the DEGs in root nodules, *Phvul*.*009G231000* was recently identified as a candidate gene for SNF using GWAS on an Andean bean diversity panel [24]. Currently, there is no functional annotation for *Phvul*.*009G231000* in Phytozome 10.3. However, *Phvul*.*009G231000* has high sequence similarity to *AT2G26190* in *Arabidopsis thaliana*, which encodes a calmodulin-binding protein. Calmodulin proteins are associated with calcium fluxes. The nodulation-signaling pathway has been reported to contain calcium-activated kinases [16]. The identification of *Phvul*.*009G231000* as a candidate gene for SNF in two studies with different approaches and genetic backgrounds provides further support for the possible role of *Phvul*.*009G231000* in SNF in common bean.

SNF is a developmentally and temporally integrated process established in early stages of plant development and continue through flowering stage until nodule senescence. The current study only focused on SNF at flowering stage, and identified genes potentially involved in explaining differences SNF rates of SA36 and SA118. Some of genes involved in N fixation before flowering, which could have contributed to observed biomass and accumulated N differences between SA36 and SA118 could have been missed. Phenotypic selection for SNF is expensive and sometimes ineffective because of environment effects on traits controlling SNF. Development of gene-based markers can circumvent these challenges. The SNPs in DEGs identified in this study can be used to develop gene-based markers to indirectly select for enhanced SNF. These markers would be more informative as they are derived from genes not only important to SNF, but also contribute to genetic variability in SNF in common bean.

## Conclusions

Genes that are differentially expressed between SA36 and SA118 under N-fixing conditions, but not under non-fixing conditions were identified. These DEGs encode various proteins including receptor kinases, TFs and transporters as well as genes with no functional annotation. Significantly enriched molecular functions in DEGs upregulated in SA36 (RIL with higher SNF rate) include purine nucleoside binding, transmembrane receptor kinase, and transport activities. The identified DEGs and their enriched molecular functions form the molecular genetic basis of the contrasting SNF phenotypes between SA36 and SA118. Genes encoding TFs identified in the current study are strong candidates for future functional studies aimed at characterizing the role of TFs in SNF to further our understanding of the gene regulatory network of SNF. In addition, the DEGs identified and data generated in the current study provide a valuable resource for developing a set of gene-based markers specific to SNF that can be used to accelerate the genetic improvement of common bean for enhanced SNF.

## Supporting information

S1 Table Statistics summary of read mapping to the common bean genome. (DOCX)

S2 Table Pearson correlation coefficients among replications under non-fixing conditions. (XLSX)

S3 Table Genes differentially expressed in leaves between SA36 and SA118 under fixing condition but were not differentially expressed under non-fixing condition. (XLSX)

S4 Table Genes differentially expressed in roots between SA36 and SA118 under fixing condition but were not differentially expressed under non-fixing condition. (XLSX)

S5 Table Genes differentially expressed in nodules between SA36 and SA118. (XLSX)

S6 Table List of single nucleotide polymorphism (SNPs) and their physical positions in genes that were differentially expressed in leaves between SA36 and SA118. (XLSX)

S7 Table List of single nucleotide polymorphism (SNPs) and their physical positions in genes that were differentially expressed in roots between SA36 and SA118. (XLSX)

S8 Table List of single nucleotide polymorphism (SNPs) and their physical positions in genes that were differentially expressed in nodules between SA36 and SA118. (XLSX)

## Abbreviations

DEG: Differentially expressed gene
RIL: Recombinant inbred line
SNF: Symbiotic nitrogen fixation
TF: Transcription factor
