# Supplementary material for: Transcriptome analysis of two recombinant inbred lines of common bean contrasting for symbiotic nitrogen fixation
Source: PLoS One. 2017 Feb 13;12(2):e0172141. doi: 10.1371/journal.pone.0172141 (PMC5305244; doi:10.1371/journal.pone.0172141)
Supplement: S1 Table — (DOCX) [file pone.0172141.s001.docx]

S1 Table. Statistics summary of read mapping to the common bean genome

| Tissue (Replication) | Total Reads | High-quality | Mapped (%) | Uniquely Mapped  (% of mapped) |
| --- | --- | --- | --- | --- |
| **Fixing Conditions** |  |  |  |  |
| SA118 Nodule (R1) | 29,746,570 | 29,297,934 | 28,193,790 (96.2) | 25,161,438 (89.2) |
| SA118 Nodule (R2) | 39,369,353 | 38,614,671 | 37,304,756 (96.6) | 34,695,243 (93.0) |
| SA118 Nodule (R3) | 30,084,472 | 28,834 719 | 27,658 997 (95.9) | 23,950,737 (86.6) |
| SA36 Nodule (R1) | 35,885,970 | 35,340,122 | 33,760,366 (95.5) | 30,790,251 (91.2) |
| SA36 Nodule (R2) | 19,758,854 | 19,675,059 | 18,975,163 (96.4) | 17,506,439 (92.3) |
| SA36 Nodule (R3) | 23,291,342 | 23,186,169 | 22,400,771 (96.6) | 21,056,138 (94.0) |
| SA36 Leaf (R1) | 22,924,623 | 22,868,500 | 21,995,986 (96.2) | 20,873,291 (94.9) |
| SA36 Leaf (R2) | 24,586,264 | 24,470,190 | 23,902,260 (97.7) | 22,578,284 (94.5) |
| SA36 Leaf (R3) | 24,208,366 | 24,160,247 | 23,602,246 (97.7) | 22,332,576 (94.6) |
| SA118 Leaf (R1) | 31,287,828 | 30,731,986 | 29,211,106 (95.1) | 27,982,601 (95.8) |
| SA118 Leaf (R2) | 32,367,954 | 31,948,215 | 31,266,813 (97.9) | 28,818,909 (92.2) |
| SA118 Leaf (R3) | 20,133,267 | 20,084,301 | 19,626,510 (97.7) | 18,061,453 (92.0) |
| SA36 Root (R1) | 32,132,215 | 31,702,339 | 31,066,482 (98.0) | 30,127,895 (97.0) |
| SA36 Root (R2) | 26,133,451 | 25,915,534 | 25,388,570 (98.0) | 24,660,032 (97.1) |
| SA36 Root (R3) | 26,740,990 | 26,626,277 | 26,098,242 (98.0) | 25,365,596 (97.2) |
| SA118 Root (R1) | 27,800,225 | 27,579,786 | 26,400,450 (95.7) | 25,705,966 (97.4) |
| SA118 Root (R2) | 22,374,226 | 22,338,255 | 21,825,866 (97.7) | 21,076,211 (96.6) |
| SA118 Root (R3) | 24,904,597 | 24,744,936 | 24,106,901 (97.4) | 24,336,187 (96.8) |
| **Non-Fixing Conditions** | | | | |
| SA36 Leaf (R1) | 26,609,927 | 26,113,636 | 25,223,395 (96.6) | 23,567,101 (93.4) |
| SA36 Leaf (R2) | 32,387,528 | 32,364,064 | 31,572,228 (97.6) | 29,658,298 (93.4) |
| SA36 Leaf (R3) | 21,454,037 | 21,408,563 | 20,975,741 (98.0) | 19,677,080 (93.8) |
| SA118 Leaf (R1) | 36,376,779 | 36,268,748 | 35,522,625 (97.9) | 33,116,973 (93.2) |
| SA118 Leaf (R2) | 41,744,529 | 41,392,746 | 40,496,211 (97.8) | 38,727,429 (95.6) |
| SA118 Leaf (R3) | 21,454,037 | 21,408,563 | 20,975,741 (98.0) | 19,677,080 (93.8) |
| SA36 Root (R1) | 34,378,160 | 34,169,430 | 33,383,738 (97.7) | 32,459,446 (97.2) |
| SA36 Root (R2) | 31,779,328 | 31,498,002 | 30,637,386 (97.3) | 29,557,415 (96.5) |
| SA36 Root (R3) | 36,758,293 | 36,138,880 | 35,410,085 (98.0) | 34,370,648 (97.1) |
| SA118 Root (R1) | 29,185,232 | 28,904,129 | 27,928,868 (96.6) | 27,012,105 (96.7) |
| SA118 Root (R2) | 30,319,711 | 29,450,519 | 28,723,193 (97.5) | 27,922,661 (97.2) |
| SA118 Root (R3) | 25,067,610 | 24,914,856 | 23,812,750 (95.6) | 23,110,723 (97.1) |
